# Transactional sex and incident HIV infection in a cohort of young women from rural South Africa

**DOI:** 10.1097/QAD.0000000000001866

**Published:** 2018-06-18

**Authors:** Kelly Kilburn, Meghna Ranganathan, Marie C.D. Stoner, James P. Hughes, Catherine MacPhail, Yaw Agyei, F. Xavier Gómez-Olivé, Kathleen Kahn, Audrey Pettifor

**Affiliations:** a Carolina Population Center, University of North Carolina, Chapel Hill, North Carolina, USA; b Department of Global Health and Development, London School of Tropical Medicine and Hygiene, London, UK; c Department of Biostatistics, University of Washington, Seattle; d Statistical Center for HIV/AIDS Research and Prevention (SCHARP), Seattle, Washington, USA; e School of Health and Society, University of Wollongong, Wollongong, New South Wales, Australia; f Wits Reproductive Health and HIV Institute; g MRC/Wits Rural Public Health and Health Transitions Research Unit (Agincourt), University of the Witwatersrand; h International Laboratory, John Hopkins University, Johannesburg, South Africa; i INDEPTH Network, Accra, Ghana.

**Keywords:** adolescent girls, incident HIV infection, South Africa, transactional sex, young women

## Abstract

**Objective:**

In sub-Saharan Africa, young women who engage in transactional sex (the exchange of sex for money or gifts) with a male partner show an elevated risk of prevalent HIV infection. We analyse longitudinal data to estimate the association between transactional sex and HIV incidence.

**Design:**

We used longitudinal data from a cohort of 2362 HIV-negative young women (aged 13–20 years) enrolled in a randomized controlled trial in rural, South Africa who were followed for up to four visits over 6 years.

**Methods:**

The effect of transactional sex on incident HIV was analysed using stratified Cox proportional hazards models and cumulative incidence curves. Risk ratios were estimated using log-binomial models to compare the effects across visits.

**Results:**

HIV incidence was higher for young women that reported transactional sex (hazard ratio 1.59, 95% confidence interval 1.02–2.19), particularly when money and/or gifts were received frequently (at least weekly) (hazard ratio 2.71, 95% confidence interval 1.44–5.12). We also find that effects were much stronger during the main trial and dissipate at the postintervention visit, despite an increase in both transactional sex and HIV.

**Conclusion:**

Transactional sex elevates the risk of HIV acquisition among young women, especially when it involves frequent exchanges of money and/or gifts. However, the effect was attenuated after the main trial, possibly due to the changing nature of transactional sex and sexual partners as women age. These findings suggest that reducing transactional sex among young women, especially during adolescence, is important for HIV prevention.

## Introduction

Among females living with HIV worldwide, 15% are aged 15–24 years and 80% live in sub-Saharan Africa (SSA) [1]. In South Africa, adolescent girls and young women (hereafter young women) aged 15–24 years have significantly higher HIV incidence compared with men of the same age (2.5 vs. 0.6%) [2–4]. This is due to increased biological and economic vulnerability of young women, along with individual risk behaviours – such as inconsistent condom use, number of partners, and age at sexual debut – that increase their risk of HIV infection [5–8].

Transactional sex is an important source of HIV risk for young women in some settings, whereby sex is exchanged for material possessions, money, and/or favours, and differentiated from formal sex work by those who participate in the exchange [6,9–11]. Reflecting social and economic roles in some settings, it is primarily men that provide and women that receive these material benefits in transactional sexual encounters [12]. Data from Demographic and Health Surveys from 12 SSA countries show that prevalence of lifetime transactional sex varies from 2 to 27%, across settings [13]. Transactional sex is driven by structural factors, including poverty, gender inequality, and lack of education [4]. It is also driven by a number of psychosocial factors, including parental and peer pressure, aspirations for social mobility, and material consumer goods, as well as romantic notions of love and security that can prompt relationships characterized by material exchange for sex [14,15]. Definitions of transactional sex have evolved over time in an attempt to prevent a tendency to conflate it with formal sex work [14,16]. Transactional sex is not considered to be formal sex work if the exchange is undertaken within the context of a relationship (no matter how temporary or ambiguous its nature); the negotiation of the terms of the exchange is neither explicit nor upfront; and those who engage in the practice differentiate their practice from formal sex work [15].

Further, to understand the role that transactional sex plays in young women's risk of HIV, it is also important to recognize that sexual relationships involving material exchange are not necessarily in and of themselves inherently risky for HIV. Given that gifts often form an integral part of courtship or expressions of affection within relationships, there might be money or gifts exchanged after a single once-off sexual act, or there may be sexual exchanges that occur within the context of adolescent romantic relationships [17]. However, under certain circumstances, transactional sex might impart greater HIV risk, such as when material gain is sometimes the only factor sustaining the relationship or the frequency at which gifts and/or money are received, resulting in young women being placed in a nonnegotiable position due to their reliance on partners [15,17].

Overall, the epidemiological evidence demonstrating the relationship between transactional sex and HIV is primarily based on cross-sectional data [11]. This has made the assessment of causality challenging, as it is difficult to assess the timing of transactional sex in relation to the acquisition of HIV. A systematic review by Wamoyi *et al.*[11] confirmed the importance of transactional sex on women's risk of HIV in SSA. Of the 14 studies that showed a positive association, only one study used a longitudinal design, revealing an important gap in the literature. The purpose of this study is to longitudinally assess the effect of transactional sex, including any exposure and by the frequency of material exchanges, on HIV incidence among a cohort of rural South African young women.

## Methods

### Data and ethics

The current article is a secondary analysis of longitudinal data of young women living in Mpumalanga province, South Africa who were enrolled in the HIV Prevention Trials Network (HPTN) 068 study. HPTN 068 was a phase III, individually randomized control trial to determine the efficacy of conditional cash transfers (CCT) to reduce the risk of HIV acquisition among young women [18]. The study recruited young women between the ages of 13–20 years enrolled in high school (grades 8–11) in the Agincourt demographic health surveillance site. Conditions for enrolment in the study included not being pregnant or married, able to read and open a post office or bank account, and living with a parent or guardian.

Young women (and their parent or guardian) were individually randomized (1 : 1) to either the treatment group (monthly cash transfer conditional on school attendance) or control group (no cash). All participants were assessed before randomization and then reassessed annually at 12, 24, and 36 months until they graduated from high school or the study ended, whichever came first. An additional ‘graduation’ visit, which only consisted of HIV and herpes simplex virus 2 (HSV-2) testing, was conducted for some young women that graduated high school before the end of the study. A trial profile with numbers at each visit has been previously published [18]. One additional visit took place 1–2 years after the end of the study (a postintervention visit) for all participants, thus young women could have up to four follow-up visits. At each visit (except the graduation visit), young women completed an audio computer-assisted self-interview (ACASI), which allows participants privacy in answering questions that are sensitive in nature, such as sexual behaviours. All visits included HIV and HSV-2 testing (if negative at the previous visit), and HIV pretest and posttest counselling.

Institutional Review Board approval for this study was obtained from the University of North Carolina at Chapel Hill and the University of the Witwatersrand Human Research Ethics Committee, South Africa, as well as the Provincial Department of Health's Research Ethics Committee.

### Sample

At baseline, 2533 young women were enrolled in the main study. Our analytical sample includes 2362 young women that were HIV negative at baseline and had at least one follow-up visit. Of the 2533 young women, 85 were excluded as they were either HIV positive or inconclusive at baseline and 82 were excluded as they did not have any follow-up visit after baseline. Censoring weights were included during the analysis of the main trial and found not to have an influence on study impacts [18].

During the main study, there were 107 seroconversions in 5031 person-years of follow-up, with an HIV incidence of 2% per year. Out of these 107 women, 22 had seroconverted at a graduation visit that only consisted of an HIV test with no corresponding ACASI survey. Those HIV results were therefore matched to the young woman's last ACASI survey completed after baseline. The median time between the last visit with an ACASI survey to the graduation HIV test for all participants was 5 months (interquartile range: 4, 6). One young woman had no follow-up survey data leaving 106 HIV positive events to use from the study period. At the postintervention visit, there were an additional 100 seroconversions for a total of 206 HIV events with 9523 person-years of follow-up.

In our analysis, we included all baseline negative HIV young women to estimate the effect of transactional sex on HIV incidence. We do not exclude sexually inactive young women because a meaningful proportion of the incident HIV infections (20%) occurred in young women who did not report any sexual activity, and we wanted to extrapolate findings to all young women. However, we also provide results among those who reported ever having sex as a sensitivity analysis in Appendix Table A1.

### Measures

The outcome variable is HIV incidence, which was determined using HIV tests conducted at baseline and each follow-up visit. HIV testing procedures included using two HIV rapid test performed in parallel [determine HIV 1/2 (Alere Medical Co, Matsudo-Shi, Chiba, Japan) and the Uni-Gold Recombigen HIV test (Trinity Biotech, Bray County, Wicklow, Ireland)]. A confirmatory test was performed using the GS HIV-1 Western Blot assay (Bio-Rad Laboratories Inc., Redmond, Washington, USA) if one or both the rapid HIV results were reactive [18].

The main exposure of transactional sex is operationalized as whether a young woman said that she felt that she had to have sex with a male partner as he gave her money or gifts. At each visit, questions from the ACASI asked about her three most recent sexual partners (including anyone with whom she had sex) in the past 12 months prior to each interview. We used the concurrent transactional data and HIV results at each visit, but for HIV events that were found during the graduation visit, the young woman's most recent survey was used. We then created a time-varying binary exposure variable for transactional sex that equals 1 if she responded ‘yes’ to transactional sex for any of these partners at that concurrent or most recent visit.

An additional categorical exposure variable was created to compare the effect of having transactional sex with a partner that gave money or gifts frequently vs. infrequently. Questions about the frequency of money and gifts were asked on different scales so we defined frequent exchanges of material items as receiving money weekly and gifts as ‘often’ or ‘always’. Infrequent exchanges of items were defined as having received money once or monthly and gifts ‘a few times’ or ‘once’. The exposure therefore has three categories: no transactional sex, transactional sex with infrequent receipt of money and/or gifts, and transactional sex with frequent receipt of money and/or gifts. We classified young women as having frequent exchanges if they had frequent exchanges with any partner, even if they reported infrequent exchanges with another partner.

Based on prior literature, we adjusted for variables that we hypothesized as confounders in the relationship between transactional sex and HIV. Time-varying controls include schooling (high school attainment or enrolled in high school), ever pregnant, intimate partner violence (IPV) (prior visit), HSV-2 status (prior visit), and per capita household consumption (prior visit). We also adjust for age of the young woman at baseline and the CCT randomization arm to account for the original trial design. When data were missing for covariates, we used the next most recent observed values as missingness in covariates was low (<5%) and a number of controls are relatively stable over visits.

Schooling is an indicator coded as 1, if the young woman had either graduated high school or was enrolled in high school at that visit. IPV is coded as 1, if the young woman experienced at least one episode of physical partner violence at the prior visit, measured using the WHO violence against women instrument for physical partner violence (six items) [19]. Per capita household consumption (prior visit) represents monthly expenditures on food and nonfood divided by the number of household members. To account for the right-skewed nature of our data, we used the log-transformation to normalize the distribution of per capita consumption.

### Analysis

To estimate the effect of time-varying transactional sex on HIV incidence, we used longitudinal data over four follow-up visits and fit an extended Cox proportional hazards model [20], stratified by grade at baseline to account for the study design whereby girls would graduate high school and out of the programme [18]. The outcome, HIV incidence, was time to a young woman's first positive HIV outcome measured at each of the yearly follow-up visits. Time was modelled in person-years starting from the date of a young woman's first negative HIV test at enrolment until the date of HIV infection or the date of censoring if she was lost to follow-up, graduated from high school, or reached the end of the study period. To visually capture the relationship between transactional sex and HIV incidence over the study period, we additionally estimated weighted cumulative incidence curves using an extended Kaplan–Meier method for time-varying exposures [21]. We generated stabilized inverse probability weights [22], which accounted for all previously defined confounders, to weight curves for each transactional sex exposure.

We then compared the main trial and postintervention periods as separate, discrete time periods to further examine the role of time (in results not shown, an interaction effect included in our Cox model between the exposure and an indicator for the postintervention visit provided the same results). Our reasoning was that by the postintervention visit, young women may have different sets of concerns or motivations as they are transitioning into young adulthood and are no longer enrolled in the cash transfer trial (note that the cash transfer did not have a significant effect on reducing transactional sex). We fit log-binomial regressions using generalized linear models to estimate risk ratios (RR) of the effect separately for the two time periods [in addition, because 22 HIV events drop from the models due to missing values of transactional sex from the most recent visit, we examined the effects of having transactional sex at any prior visit using the same log-binomial models. Results are robust to this definition of exposure (Table A3)]. Models were adjusted for person-years of exposure (from her first negative HIV test) in addition to all previously defined confounders. As a robustness analysis, we modelled Kaplan–Meier weighted cumulative incidence curves again but used young women's age (in discrete years) as analysis time.

To test for significant differences between categories of transactional sex (receives money and/or gifts infrequently vs. frequently), we used a Wald test with a chi^2^ distribution. All analyses were performed using Stata 14.2 (Stata Corp., College Station, Texas, USA) and the validity of the proportional hazards assumption was evaluated using tests based on Schoenfeld residuals.

## Results

Table 1 displays the baseline characteristics of our sample for the entire sample. Among the entire sample at baseline, young women had a median age of 15 years, 20% were single or double orphans, and 34% had ever repeated a grade (all were enrolled at baseline per the selection criteria). Only a quarter of young women reported ever having sex and the median age of first sex was 16 years old. With a low proportion of sexually active participants among the entire sample, risky sexual behaviours such as transactional sex and having a partner five or more years older were also low (3.6 and 5.6%, respectively), whereas 8% had ever been pregnant and 4% had prevalent HSV-2 infection.

Table 2 reports the hazard ratios for the effects of transactional sex on HIV incidence for the entire sample. Compared with those who did not report transactional sex, young women that reported any transactional sex during the same (or most recent) visit, as their HIV test have a higher hazard of HIV [hazard ratio 1.50, 95% confidence interval (CI) 1.02–2.19] after adjusting for confounders. This estimate is even higher if they had transactional sex with a partner that frequently gave them money (hazard ratio 2.71, 95% CI 1.44–5.12). In contrast, there is no significant effect for young women that had transactional sex with a partner that gave them money and/or gifts infrequently. Sensitivity analysis demonstrates that the effects of transactional sex by frequency also hold among the sample of sexually active young women (Table A1).

**Fig. 1:**
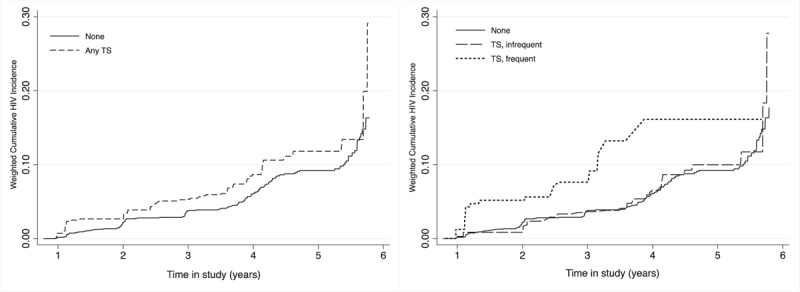
Cumulative HIV incidence by transactional sex exposures.

Results look similar using weighted cumulative incidence curves (Fig. 1). However, although transactional sex with frequent exchanges is clearly driving the relationship, after year 4, the cumulative HIV risk stops increasing, corresponding to the postintervention study period. A risk table shows that the number at risk for any transactional sex is increasing across visits but the distribution is trending towards infrequent exchanges (Table 3). Descriptively, we found that the proportion of women engaging in any transactional sex increases at the postintervention visit (17.3%) compared with the main trial (9.5%) as does HIV (incidence of 2.2 vs. 5.6%) (Table 4). However, the proportion having transactional sex with a partner who provides frequent money and/or gifts declines at the postintervention visit (1.2%) compared with during the main trial (4.7%) (Appendix Table A1 provides a further breakdown by each study visit). Therefore, by the post-intervention visit (Visit 4), while the total number at risk is still large, only 22 young women are at risk in the frequent exchanges category (Table 3).

Weighted failure curves estimated using the extended Kaplan–Meier method. Inverse probability of treatment weights based on the following covariates: baseline age, CCT study arm, graduated or enrolled in high school, ever pregnant, any intimate partner violence at previous visit, HSV-2 status at previous visit, and log household consumption at previous visit. CCT, conditional cash transfer; HSV-2, herpes simplex virus-2, TS, transactional sex.

Next, we examined the role of time on the relationship between transactional sex and HIV by splitting the sample into the two discrete study periods, during the main trial (Visits 1–3) and the postintervention visit (Visit 4). Results from Table 4 also indicate a shifting relationship between transactional sex and HIV. Notably, evidence of statistical significance between transactional sex and HIV is lacking at the postintervention visit. During the main trial, there is elevated risk of HIV for young women that had any transactional sex (RR 2.02, 95% CI 1.19–3.43), and this effect is robust for the categorical exposure. For the postintervention visit, however, there is no effect for either transactional sex exposure. In addition, there are no HIV events for young women that report frequent exchanges at the postintervention visit, so effects are the same for the binary and categorical exposures. Sensitivity analysis for the sexually active group shows a similar pattern of results but weaker effects (Table A2). Furthermore, we compared young women that were reinterviewed at the postintervention visit and those that were not to examine whether the postintervention results could be driven by attrition of young women that were ‘riskier’ in terms of sexual behaviours. We did not find evidence that not being reinterviewed (*n* = 461) was associated with transactional sex, the frequency of exchanges, or HIV acquisition (results not shown).

**Fig. 2:**
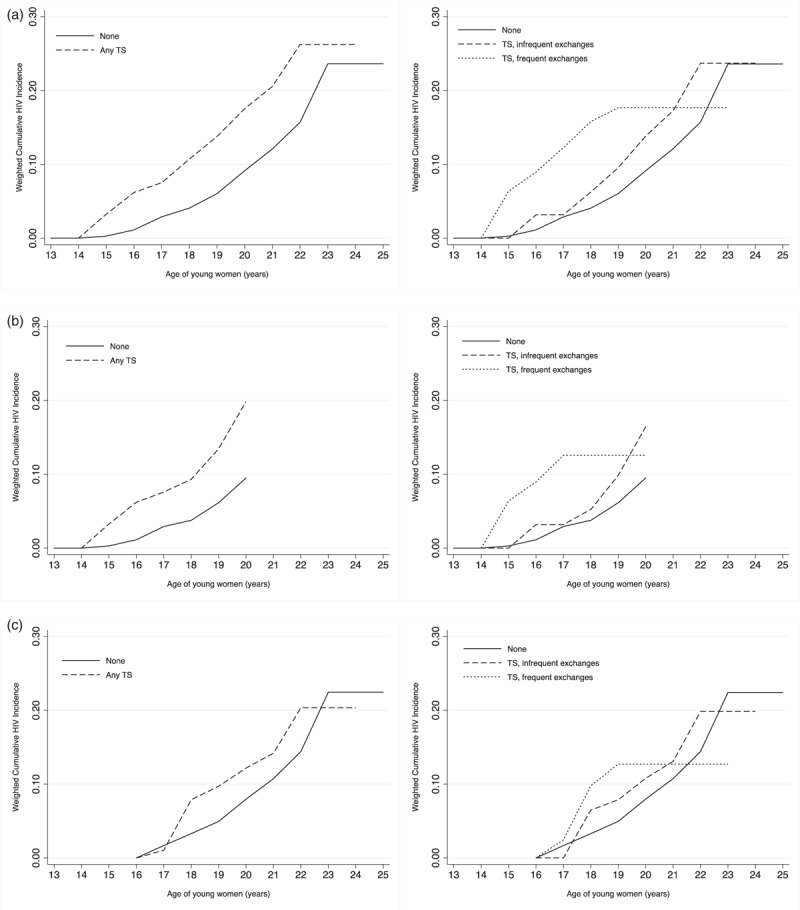
Cumulative HIV incidence by transactional sex exposures with age in years as analysis time.

Lastly, using the extended Kaplan–Meier method, we estimated weighted cumulative incidence curves with age as the time unit (Fig. 2). Across the entire sample, the relationship between HIV risk and transactional sex is stronger among the younger adolescent girls, whereas for the women in their 20s, HIV risk starts to level out (Fig. 2a). The cumulative HIV incidence also rises more rapidly for those who engage in transactional sex with frequent exchanges (Fig. 2b), especially among the group of girls that were the youngest at baseline (Fig. 2b) compared with the young women that started the study at 16 or older (Fig. 2c).

Weighted curves estimated using the extended Kaplan–Meier method. Inverse probability of treatment weights based on the following covariates: baseline age, CCT study arm, graduated or enrolled in high school, ever pregnant, any intimate partner violence at previous visit, HSV-2 status at previous visit, and log household consumption at previous visit. (a) Full sample (*N* = 2362). (b) Young women aged under 16 at baseline (*N* = 1256). (c) Young women aged 16 and over at baseline (*N* = 1106). CCT, conditional cash transfer; HSV-2, herpes simplex virus-2, TS, transactional sex.

## Discussion

Our results show that transactional sex is associated with HIV incidence for young women and lends support to the only other study measuring the relationship between transactional sex and HIV incidence in young women in South Africa [6]. We also find that the effect is driven by relationships in which a sexual partner provides money and/or gifts at least weekly, suggesting that the inherent risk of transactional sex for HIV increases with the frequency by which the young woman receives material items from her partner.

A plausible reason for this may be that young women in these partnerships simply have more sexual encounters with male partners that are transactional in nature, thereby increasing their exposure to HIV and sexually transmitted infections. In addition, an imbalance in power dynamics may be more acute in these partnerships in which men are providing money/gifts more frequently [23]. Young women may also feel less able to leave such relationships if they rely on the money to get by or maintain a lifestyle. This aligns with research in Kenya by Luke who showed that the amount of money received by young women was significantly associated with a higher frequency of sex and inconsistent condom use, even after controlling for measures of love and commitment [24]. In our study, among young women that had transactional sex, we find higher rates of IPV and lower levels of sexual relationship power (Table A5). This offers supportive evidence for this conclusion. Nonetheless, condom use is reported at high levels for both groups.

Further analysis revealed that the risk of transactional sex differed also by the time period and age of exposure. At the postintervention visit, transactional sex did not predict HIV incidence despite higher rates of both transactional sex and HIV. We attribute this discrepancy to changes associated with young women transitioning into young adulthood and maturing. This is because at the postintervention visit when women are older (65% are over 19), HIV risk is equally high among those who engage in transactional and those who do not (incidence of around 6% each). In addition, the association between HIV risk and transactional sex appears to be stronger during younger ages. Figure 2 shows that cumulative risk of HIV increases more rapidly for those engaging in transactional sex at younger ages (roughly before age 20), but at older ages, cumulative risk is increasing faster for those not engaging in transactional sex.

There are several hypotheses for the changing nature of the relationship between transactional sex and incident HIV infection as young women get older. First, the interaction between age and sexual activity is important. For one, during their 20s, most of the cohort is sexually active so a higher HIV prevalence in the pool of same-age sexual partners could contribute to an overall increase in their HIV risk. During adolescence, however, it might be a riskier type of girl who is sexually active and so other risk behaviours may tend to cluster (e.g. engaging in age-disparate sex and transactional sex). This results in a concentration of HIV infection in younger ages among those that exhibit risky sexual behaviour. This would align with evidence that a predisposition to risk-taking interacts with the adolescent developmental process, whereby younger adolescents are more prone to risk-taking as the brain's prefrontal cortex (associated with rationality and impulse control) is still developing [25].

Second, despite the higher prevalence of age-disparate partnerships and transactional sex at the postintervention visit (among young women mostly in their 20s), transactional sex with frequent exchanges of money and/or gifts is uncommon. This decrease in the frequency of material items might be as older women are more financially independent and hence have more resources, are in longer term relationships and so do not expect gifts as often, or as they have become better at negotiating condom use with age. Therefore, as young women age and transition into a different stage of life, these findings may reflect changes in their primary motivations around obtaining items to improve their status and to satisfy feelings of needing to belong [26]. More research is needed to understand the evolving nature of transactional sex as young women age and transition into adulthood.

The current study adds to the literature by following a cohort of young women from adolescence to young adulthood to assess the relationship between transactional sex and incident HIV. The analysis is strengthened by the use of longitudinal data with HIV biomarkers. Our study is limited, however, in a few ways. Although we are able to examine frequency of material exchanges, we do not know if this directly corresponded to the frequency of sexual exchanges; thus our measure is a proxy for the intensity of transactional sex relationships. Ideally, we would also have restricted our analysis to women that reported being sexually active, but given sample size constraints and an outcome with rare events, we would lose power and confidence to estimate meaningful effects.

Further, as sexual activity was self-reported, we expect that underreporting was a likely issue, especially as we found incident HIV infections among young women that do not report ever having sex. However, there is no reason to suspect that misreporting sexual behaviour is associated with HIV status in the analysis, as the HIV tests occurred after young women answered the ACASI questions. In fact, as all of the incident HIV cases that were nonsexually active are defined as no transactional sex, our results may actually underestimate the strength of the association. When we examined an exposure of ever transactional sex during the study, for instance, we find stronger relationships during the main trial (Table A4). Nonetheless, we find that our main results are robust to a number of checks. First, effects are similar among the sexually active subsample (Tables A2 and A3). Moreover, we examined whether the 5% missingness in our exposure from those who report sexual activity biased our results (22 HIV events were lost in our complete cases analysis). After replacing all missing values as either 0 or 1 and estimating the minimum and maximum bounds, respectively, we find that hazard ratios are almost unchanged (CIs are slightly wider for minimum bound). As more than 10% of these missing cases reported transactional sex at least once before, our results likely approximate the true effect assuming a similar proportion of missing were exposed.

### Conclusion

Despite the declining rate of new HIV infections among young people aged 15–24, poor young women are still disproportionately affected by the epidemic in SSA. Their economic vulnerability interacts with gender power imbalances in sexual relationships to increase their risk of HIV. Both adolescent girls and young women are recognized as a prevention priority, but with increasing evidence on how differently the adolescent brain works, interventions to reduce young women's HIV risk should be designed with respect to developmental stages. Our findings reveal the need to consider the changing dynamics (i.e. economic, behavioural, and psychosocial) that influence HIV risk of adolescent girls and young women as they transition to adulthood. From a research perspective, more evidence is needed on the pathways underlying the transactional sex and HIV relationship in addition to a better understanding of the changing motivations for transactional sex as women age. This evidence will help in developing targeted strategies to reach those at-risk young women and reduce their vulnerability to HIV acquisition.

## Acknowledgements

Funding support for the HPTN was provided by the National Institute of Allergy and Infectious Diseases (NIAID), the National Institute of Mental Health (NIMH), and the National Institute on Drug Abuse (NIDA) of the National Institutes of Health [NIH; award numbers UM1AI068619 (HPTN Leadership and Operations Center), UM1AI068617 (HPTN Statistical and Data Management Center), and UM1AI068613 (HPTN Laboratory Center)]. The study was also funded under R01MH110186, R01MH087118, and R24 HD050924 to the Carolina Population Center. Research reported in this publication was also supported by the NIAID of the NIH (Award Number T32AI007001). Additional funding was provided by the Division of Intramural Research, NIAID, and NIH. The Agincourt Health and Socio-Demographic Surveillance System is supported by the University of the Witwatersrand, the Medical Research Council, South Africa and the Wellcome Trust, UK (grants 058893/Z/99/A; 069683/Z/02/Z; 085477/Z/08/Z; and 085477/B/08/Z). The content is solely the responsibility of the authors and does not necessarily represent the official views of the NIH.

Role of funding source: The funder of the study had no role in study design, data collection, data analysis, data interpretation, or writing of the report. The corresponding author had full access to all the data in the study and had final responsibility for the decision to submit for publication.

Authors’ contributions: K.K., M.C.D.S., and M.R. contributed to data analysis, data interpretation, and writing of this article. A.P. contributed to study design, development of data collection instruments and the protocol, study oversight and implementation, data interpretation, data analysis, and editing of this article. C.M. and K.K. contributed to study design, development of data collection instruments and the protocol, study oversight and implementation, and editing of this article. J.P.H. contributed to study design, development of the protocol, data interpretation, and editing of this article. X.G.-O. contributed to study implementation and oversight, data interpretation, and editing of the article. Y.A. contributed to oversight of laboratory testing, data interpretation, and editing of the article.

### Conflicts of interest

There are no conflicts of interest.

## Supplementary Material

Supplemental Digital Content

## Figures and Tables

**Table 1:** Baseline characteristics of study participants (*N* = 2362).

	*N* (%) or median (IQR)
Age (years)	15 (14, 17)
Orphan (double or single)	468 (20.0%)
Ever repeated a grade	800 (33.9%)
Household monthly per capita expenditure (Rand)	289 (185, 478)
CCT intervention arm	1215 (51.4%)
Ever sex	618 (26.2%)
Age at first sex (sexually active)	16 (14, 16)
Condom use at last sex (sexually active)	426 (69.5%)
High relationship power (sexually active)	227 (37.9%)
Any transactional sex past 12 months	82 (3.6%)
Ever pregnant	192 (8.1%)
Prevalent HSV-2 infection	90 (3.8%)
Older partner (5+ years older)	129 (5.6%)

Number of missing values: age, 0; orphan, 19; repeated grades, 0; per capita expenditure, 1; CCT arm, 0; transactional sex, 105; ever sex, 3; age at first sex, 10; condom use, 5; low relationship power, 19; ever pregnant, 0; HSV-2, 3; older partner 38. CCT, conditional cash transfers; HSV-2, herpes simplex virus; IQR, interquartile range.

**Table 2:** Hazard ratios for the effect of transactional sex on HIV incidence in a cohort of young women from HIV Prevention Trials Network 068.

	No. HIV events	Person-years	HR (95% CI)
Transactional sex, binary	Total = 184		
None	143	7709	1
Any	41	1273	1.50^**^ (1.02–2.19)
Transactional sex, categorical			
None	143	7709	1
Infrequently receives money/gifts	29	971	1.24 (0.81–1.91)
Frequently receives money/gifts	12	302	2.71^***^ (1.44–5.12)
Chi^2^ test for equality of effects comparing infrequent with frequent			*P* = 0.03

Hazard ratios (HRs) from Cox proportional hazards model, stratified by grade. Models adjusted for: baseline age, CCT study arm, graduated or enrolled in high school, ever pregnant, any IPV at last visit, HSV-2 status at last visit, and log household consumption at last visit. CCT, conditional cash transfer; CI, confidence interval; HSV-2, herpes simplex virus-2, IPV, intimate partner violence.

^**^P < 0.05.

^***^P < 0.01.

**Table 3:** Number of young women at risk over study visits.

	Main trial			Postintervention
	Visit 1	Visit 2	Visit 3	Visit 4
	12-month	24-month	36-month	48–60-month
Total at risk	2361	2206	2005	1827
Transactional sex, binary				
None	2037	1852	1584	1381
Any	222	254	301	314
Transactional sex, categorical				
None	2037	1852	1584	1381
Infrequent	119	157	231	292
Frequent	103	97	70	22

Estimates from Kaplan–Meier failure curves with time modelled by discrete visits. The total at risk represent the entire sample at still at risk and do not necessarily equal the sum of those at risk across transactional sex categories due to missingness in the exposure.

**Table 4:** Risk and risk ratios for the effects of transactional sex on HIV incidence by main trial vs. postintervention.

	During the main trial (3 visits)				Postintervention (1 visit)			
	*N* = 4913 observations (2303 individuals)				*N* = 1901 observations and individuals			
	*N* (%)	No. HIV events	Risk (%)	Risk ratio^a^ (95% CI)	*N* (%)	No. HIV events	Risk (%)	Risk ratio (95% CI)
Transactional sex, any								
None		69	1.7	1		74	5.5	1
Any	466 (9.5%)	23	5.0	2.02^**^ (1.19–3.43)	328 (17.3%)	18	5.8	0.98 (0.58–1.66)
Transactional sex, categorical								
None		69	1.7	1		74	5.5	1
Infrequently received money/gifts	234 (4.8%)	11	4.8	1.97^***^ (1.05–3.71)	305 (16.1%)	18	5.8	0.98 (0.58–1.66)
Frequently received money/gifts	232 (4.7%)	12	5.3	2.08^***^ (1.03–4.21)	23 (1.2%)	0	–	–
Chi^2^ test for equality of effects comparing infrequent with frequent				*P* = 0.90				*–*

Log-binomial regressions with robust standard errors. Models adjusted for baseline age, CCT study arm, person-years of exposure, graduated or enrolled in high school, ever pregnant, any IPV at last visit, HSV-2 status at last visit, and log household baseline consumption. CCT, conditional cash transfer; CI, confidence interval; HSV-2, herpes simplex virus-2, IPV, intimate partner violence.

^a^Adjusted for multiple visits by individuals.

^**^P < 0.01.

^***^P < 0.05.
