# Supplementary material for: Transactional sex and incident HIV infection in a cohort of young women from rural South Africa
Source: AIDS. 2018 Jun 18;32(12):1669–77. doi: 10.1097/QAD.0000000000001866 (PMC6082595; doi:10.1097/QAD.0000000000001866)
Supplement: Supplemental Digital Content [file aids-32-1669-s001.doc]

**Supplementary Material: Appendix**

Table A1. Summary statistics for transactional sex exposures and HIV across visits

|  | **During the main trial (3 visits)** | | | **Post-intervention (1 visit)** | | |
| --- | --- | --- | --- | --- | --- | --- |
|  | **12-month** | **24-month** | **36-month** |  | **48 to 60-month** |  |
| Transactional sex, any | 182 (8.2%) | 178 (9.8%) | 106 (12.1%) |  | 328 (17.3%) |  |
| Transactional sex, categorical |  |  |  |  |  |  |
| Infrequently received money/gifts | 91 (4.1%) | 92 (5.1%) | 51 (5.8%) |  | 305 (16.1%) |  |
| Frequently received money/gifts | 91 (4.1%) | 86 (4.7%) | 55 (6.3%) |  | 23 (1.2%) |  |
| *N interviewed* | 2,214 | 1,824 | 875 |  | 1,901 |  |
| HIV incidence | 39 (1.8%) | 40 (2.2%) | 28 (3.3%) |  | 100 (5.6%) |  |
| *N at risk* | 2,214 | 1,800 | 846 |  | 1,782 |  |

Notes: The number of participants interviewed at each visit during the main trial corresponds to the study design where enrolled young women in upper grades were graduating out of high school and the program. The post-intervention visit was for all young women that had been enrolled in the study.

Table A2. Hazard ratios for the effect of transactional sex on HIV incidence in a cohort of young women from HPTN 068 (sexually active)

|  | **No.**  **HIV events** | **Person**  **years** | **HR**  **(95% CI)** |
| --- | --- | --- | --- |
| **Transactional sex, any** | Total=140 |  |  |
| None | 99 | 3648 | 1 |
| Any | 41 | 1273 | 1·27  (0·87 - 1·86) |
| **Transactional sex, categorical** |  |  |  |
| None | 99 | 3648 | 1 |
| Infrequently receives money/gifts | 29 | 971 | 1·07  (0·69 - 1·65) |
| Frequently receives money/gifts | 12 | 302 | 2·20**  (1·16 - 4·17) |
| Test for equality of effects |  |  | *p*=0·05 |

Notes: *p<0·1; ** *p*<0·05 ***p<0·01. Estimates are for the sexually active sample from the cohort of baseline HIV negative young women. HRs from Cox proportional hazards model, stratified by grade, and with standard errors adjusted for clustering at the individual level. Controls for the adjusted models include: baseline age, CCT study arm, graduated or enrolled in high school, ever pregnant, any IPV at last visit, HSV-2 status at last visit, and log household consumption at last visit.

Table A3. Risk and risk ratios for the effects of transactional sex on HIV incidence by follow-up visits (sexually active)

|  | **During the main trial (3 visits)** | | | **Post-intervention (1 visit)** | | |
| --- | --- | --- | --- | --- | --- | --- |
|  | No. events | Risk (%) | Risk Ratio **^1^** (95% CI) | No. events | Risk (%) | Risk Ratio (95% CI) |
| **Transactional sex at last visit, binary** |  |  |  |  |  |  |
| None | 45 | 3·2 | 1 | 54 | 6·4 | 1 |
| Any | 23 | 5·0 | 1·53*  (0·93 - 2·52) | 18 | 5·8 | 0·89  (0·52 - 1·52) |
| **Transactional sex at last visit, categorical** |  |  |  |  |  |  |
| None | 45 | 3·2 | 1 | 54 | 6·4 | 1 |
| Infrequently receives money/gifts | 11 | 4·8 | 1·48  (0·79 - 2·76) | 18 | 5·8 | 0·89  (0·52 - 1·52) |
| Frequently receives money/gifts | 12 | 5·3 | 1·59  (0·82 - 3·06) | 0 | -- | -- |
| Chi2 test for equality of effects comparing infrequent to frequent |  |  | *p*=0·87 |  |  | *--* |

Notes: *p<0·1; ** *p*<0·05 ***p<0·01. Log-binomial regressions with robust standard errors. Estimates are for the sexually active sample from the cohort of baseline HIV negative young women. Controls include: baseline age, CCT study arm, person-years of exposure, graduated or enrolled in high school, ever pregnant, any IPV at last visit, HSV-2 status at last visit, and log household baseline consumption. **^1^**Adjusted for clustering for multiple visits by individuals

Table A4. Risk and risk ratios for the effects of transactional sex (ever during study) on HIV incidence (full sample)

|  | **During the main trial (3 visits)** | | | **Post-intervention (1 visit)** | | |
| --- | --- | --- | --- | --- | --- | --- |
|  | **No. HIV events** | **Risk (%)** | **Risk Ratio ^1^ (95% CI)** | **No. HIV events** | **Risk (%)** | **Risk Ratio (95% CI)** |
| **Transactional sex during any prior visit, any** |  |  |  |  |  |  |
| None | 68 | 1·6 | 1 | 71 | 5·6 | 1 |
| Any | 38 | 5·8 | 2·17***  (1·35 - 3·49) | 29 | 5·7 | 0·90  (0·57 - 1·41) |
| **Transactional sex during any prior visit, categorical** |  |  |  |  |  |  |
| None | 68 | 1·6 | 1 | 71 | 5·6 | 1 |
| Infrequently receives money/gifts | 17 | 5·5 | 2·12***  (1·21 - 3·72) | 20 | 6·2 | 0·99  (0·60 - 1·64) |
| Frequently receives money/gifts | 21 | 6·1 | 2·21***  (1·25 - 3·92) | 9 | 4·8 | 0·74  (0·37 - 1·47) |
| Chi2 test for equality of effects comparing infrequent to frequent |  |  | *p*=0·89 |  |  | *p*=0·45 |

Notes: *p<0·1; ** *p*<0·05 ***p<0·01. Log-binomial regressions with robust standard errors. Estimates are for the full sample from the cohort of baseline HIV negative young women. Controls include: baseline age, CCT study arm, person-years of exposure, graduated or enrolled in high school, ever pregnant, any IPV at last visit, HSV-2 status at last visit, and log household baseline consumption. **^1^**Adjusted for clustering for multiple visits by individuals

Table A5. Demographics by whether young women engaged in transactional sex by study visits

|  | **During the main trial (Visits 1-3)** | | **Post-intervention (Visit 4)** | |
| --- | --- | --- | --- | --- |
|  | Any transactional sex | | Any transactional sex | |
|  | Yes | No | Yes | No |
| Age | 18 (17, 19) | 17 (16, 18) | 20 (19, 21) | 20 (19, 21) |
| Condom use at last sex | 76·3% | 72·0% | 79·7% | 78·1% |
| High sexual relationship power (sexually active) | 38·1% | 52·5% | 49·7% | 58·3% |
| Ever pregnant | 53·4% | 12·5% | 51·2% | 34·7% |
| Older partner (5+ years older) | 26·8% | 6·0% | 39·8% | 20·1% |
| Intimate partner violence | 37·5% | 21·1% | 18·5% | 7·4% |
